# Validity of administrative data in recording sepsis: a systematic review

**DOI:** 10.1186/s13054-015-0847-3

**Published:** 2015-04-06

**Authors:** Rachel J Jolley, Keri Jo Sawka, Dean W Yergens, Hude Quan, Nathalie Jetté, Christopher J Doig

**Affiliations:** Department of Community Health Sciences, Cumming School of Medicine, University of Calgary, 3rd Floor TRW Building, 3280 Hospital Drive NW, T2N 4Z6 Calgary, AB Canada; Snyder Institute for Chronic Diseases, University of Calgary, HRIC 4AA08, 3280 Hospital Drive NW, T2N 4N1 Calgary, AB Canada; Department of Clinical Neurosciences, Cumming School of Medicine, University of Calgary, Administration Office: Room 1195 - Foothills Hospital 1403 - 29 Street NW, T2N 2T9 Calgary, AB Canada; O’Brien Institute for Public Health, Cumming School of Medicine, University of Calgary, 3rd Floor TRW Building, 3280, Hospital Drive NW, T2N 4Z6 Calgary, AB Canada; Hotchkiss Brain Institute, Cumming School of Medicine, University of Calgary, Health Research Innovation Centre Room 1A10, 3330 Hospital Drive NW, T2N 4N1 Calgary, AB Canada; Department of Critical Care Medicine, Cumming School of Medicine, University of Calgary, Foothills Medical Centre, McCaig Tower Ground Floor, ICU Administration, 3134 Hospital Drive NW, T2N 5A1 Calgary, AB Canada

## Abstract

**Introduction:**

Administrative health data have been used to study sepsis in large population-based studies. The validity of these study findings depends largely on the quality of the administrative data source and the validity of the case definition used. We systematically reviewed the literature to assess the validity of case definitions of sepsis used with administrative data.

**Methods:**

Embase and MEDLINE were searched for published articles with International Classification of Diseases (ICD) coded data used to define sepsis. Abstracts and full-text articles were reviewed in duplicate. Data were abstracted from all eligible full-text articles, including ICD-9- and/or ICD-10-based case definitions, sensitivity (Sn), specificity (Sp), positive predictive value (PPV) and negative predictive value (NPV).

**Results:**

Of 2,317 individual studies identified, 12 full-text articles met all eligibility criteria. A total of 38 sepsis case definitions were tested, which included over 130 different ICD codes. The most common ICD-9 codes were 038.x, 790.7 and 995.92, and the most common ICD-10 codes were A40.x and A41.x. The PPV was reported in ten studies and ranged from 5.6% to 100%, with a median of 50%. Other tests of diagnostic accuracy were reported only in some studies. Sn ranged from 5.9% to 82.3%; Sp ranged from 78.3% to 100%; and NPV ranged from 62.1% to 99.7%.

**Conclusions:**

The validity of administrative data in recording sepsis varied substantially across individual studies and ICD definitions. Our work may serve as a reference point for consensus towards an improved and harmonized ICD-coded definition of sepsis.

**Electronic supplementary material:**

The online version of this article (doi:10.1186/s13054-015-0847-3) contains supplementary material, which is available to authorized users.

## Introduction

Sepsis is a life-threatening condition associated with a high mortality rate, significant health care costs and long-term consequences [1-3]. It is characterized by a spectrum of severity from mild acute organ dysfunction to multi-organ failure with complex pathophysiologic processes. Differentiating sepsis as a cause of multiple organ dysfunction syndrome from other acute systemic inflammatory conditions can be difficult [4].

Many large-scale studies have relied on administrative data to identify patients with sepsis [1,2]. Examples of administrative data include hospital discharge data, emergency visit data, physician claims and hospital insurance claims data. These data are advantageous, as they are readily available and reasonably inexpensive and can include a large cohort of patients, control for some confounders such as chronic disease [5] and include individual outcomes [6]. Many times, these data code diseases using the World Health Organization International Classification of Diseases (ICD) codes [7]. The most recent version of the ICD manual in use is the tenth revision, or ICD-10. This manual exists alongside country modifications such as ICD-10-CA (the Canadian edition) and ICD-10-AM (the Australian Modification). As well, a modification of the ICD-9 version (ICD-9-CM) is still being used in a number of countries, such as the United States and Italy [8].

Prior to 1992, there was a lack of consensus regarding clinical criteria and definitions for sepsis and related conditions. The Centers for Disease Control and Prevention (CDC) reported sepsis admissions using administrative data in which the term *septicemia*, referring to the presence and spread of microorganisms via circulating blood [9], was used as a clinical case definition and did not fully incorporate the spectrum of illness that was later defined in more detail by the 1992 American College of Chest Physicians and Society of Critical Care Medicine (ACCP/SCCM) Consensus Conference clinical definitions [10].

Angus *et al.* [1] performed a large-scale, multi-centre epidemiological study in which they implemented the identification of patients with severe sepsis using an ICD-9-based algorithm that required evidence of both an infection and new-onset organ dysfunction during a single hospitalization, thereafter described as the Angus implementation coding scheme. The Angus implementation is one of the most well-known and highly cited implementations of an ICD-coded case definition for sepsis. This definition was originally validated by the authors through a comparison of aggregate data showing hospital incidence rates and patient characteristics of the cohorts captured through the ICD-9-CM algorithm versus a previous cohort captured through a prospective study of patients with sepsis by Sands *et al.* [11]. A recent study [12] validated the Angus implementation and another well-known algorithm known as the Martin implementation [2] using a reference standard based on physician-based medical chart review. The Angus implementation was reported as having a moderate to low sensitivity (Sn) of 50.3% and a positive predictive value (PPV) of 70.7%, whereas the Martin implementation had a very low Sn of 16.8% but a high PPV of 97.6%. As such, they concluded that a population of patients with severe sepsis could be captured through administrative data using the Angus case definition, but that cases would be underestimated. Studies that examined the performance of ICD coding algorithms to identify other conditions have also highlighted the great variability that exists when multiple codes are used to define a specific condition [13,14].

The accurate identification of cases of sepsis using ICD-coded administrative data for use in health services research is paramount especially if examining complex diseases such as sepsis, where burden of disease and costs of care are very high. There is currently no consensus regarding which ICD-9 or ICD-10 codes should be used to define sepsis in administrative data. A reasonable step towards the harmonization of an ICD-based definition for sepsis is to examine the literature and report the validity of published ICD-coded case definitions in administrative data.

## Material and methods

### Search strategy

We applied a modification of the search strategy methodology of St Germaine-Smith *et al*. [14]. Using the Ovid interface, we conducted searches in MEDLINE and Embase for publications published between 1992 (based on the 1992 publication date of the establishment of definition criteria for sepsis/severe sepsis by ACCP/SCCM) and 15 September 2014, applying ‘humans’ and ‘English language’ filters. In order to identify studies assessing the diagnostic accuracy of ICD codes for identifying sepsis, the Boolean operator ‘AND’ was used to combine three search concepts: sepsis, coding and validity. Articles concerning sepsis were sought using the Boolean operator ‘OR’ to combine the Medical Subject Headings (MeSH) term ‘sepsis’ and Emtree terms relevant to the condition of sepsis, including ‘severe sepsis’ and ‘septic shock’. Articles concerning the concept of coding were sought using the Boolean operator ‘OR’ to combine the MeSH terms and keyword searches for the following terms: ‘administrative data’, ‘hospital discharge data’, ‘ICD-9’, ‘ICD-10’, ‘ICD-9xM’ or ‘ICD-10xM’ (country versions), ‘medical record’, ‘health information’, ‘surveillance’, ‘physician claims’, ‘claims’, ‘hospital discharge’, ‘coding’ and ‘codes’. Articles concerning validity were sought using Boolean operator ‘OR’ to combine the MeSH and keyword searches for the terms ‘validity’, ‘validation’, ‘case definition’, ‘algorithm’, ‘agreement’, ‘accuracy’, ‘sensitivity’, ‘specificity’, ‘positive predictive value’ and ‘negative predictive value’ (Additional file 1).

### Study inclusion

To be eligible for inclusion, articles had to compare the accuracy of ICD-9 or ICD-10 codes for sepsis, severe sepsis or septic shock in an administrative database to a reference standard and report at least one of Sn, specificity (Sp), PPV or negative predictive value (NPV). For comparison purposes, studies identified in the search that validated an ICD-coded definition without reporting any diagnostic accuracy measures were excluded. The following diagnostic accuracy measures were abstracted, if provided, from each study: Sn, Sp, PPV and NPV. All bibliographical references were imported into a custom-written Java software application [15] for improved reference management and data collection. This software, called Synthesis, is described in more detail elsewhere [16]. The title and abstract of each citation identified were screened in duplicate for eligibility by two reviewers (RJJ and KJS). Any article selected as meeting eligibility criteria by either or both reviewers was then retrieved and reviewed by the same two authors for eligibility criteria. Articles excluded based on title and abstract with reasons for exclusion are given in Additional file 2. To determine inter-rater agreement, the Cohen’s κ statistic was calculated at both the title and abstract review stage and in the full-text article review stage. All articles for which there was inter-rater discord at the abstract review stage went on to full-text review. Any full-text articles for which there was inter-rater discord were reviewed a second time, and further disagreements about study eligibility at the full-text review stage were resolved through discussion until full consensus was obtained.

### Data extraction and quality assessment

One author (RJJ) abstracted data from included studies using the standardized abstraction form, including country location of study, years of data collection, validation database, sample size and type of sample population. The validated ICD codes and algorithms, diagnostic field position and ICD version used from each study were recorded along with Sn, Sp, NPV and PPV. The authors calculated Sn or Sp in cases where these values were not reported but raw data were available to calculate them.

The included studies were assessed for quality by two reviewers, (KJS and RJJ), using a standardized validation study quality checklist adapted from Benchimol *et al.* [17]. In instances where it was unclear whether a checklist item was fulfilled by the study, it was marked as uncertain. Any discrepancies between the two reviewers were resolved through discussion. Studies included were published in peer-reviewed journals; therefore, it was not necessary to obtain patient consent. This study was reviewed and approved by the Conjoint Health Research Ethics Board at the University of Calgary.

## Results

### Study characteristics

**Figure 1:**
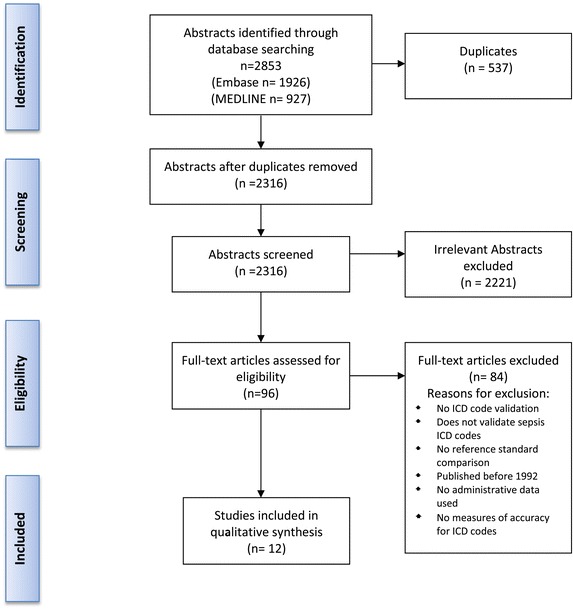
**Flow diagram for study screening and article inclusion.** ICD, International Classification of Diseases.

Of 2,317 abstracts reviewed, 96 fulfilled eligibility criteria for full-text review. Amongst these articles, the κ score for inter-rater agreement was 0.87, resulting in near-perfect agreement [18]. Twelve articles met all eligibility criteria and were included in the study [12,19-29] (Figure 1). The characteristics of the studies are shown in Table 1. All 12 studies examined hospital discharge abstract data (also called ‘inpatient administrative health data’ or ‘inpatient claims administrative dataset’). Eight of the twelve studies were performed in the United States [12,19,21,23,25,27-29], one in Australia [22], one in Denmark [24], one in Sweden [20] and one in Canada [26]. Publication dates ranged from 1998 to 2014. Seven studies examined ICD-9-CM codes, one examined only ICD-9, one examined both ICD-9 and ICD-10 codes, one study examined ICD-10, one study examined the ICD-10 Danish version and one study examined ICD-10-AM (Australian Modification) codes. The studies varied considerably in sample size (ranging from 34 to 4,181) and had heterogeneity in patients studied, including highly selective populations (rheumatoid arthritis) or sepsis clinical trial patients, to intensive care unit (ICU)-specific, general medical patients or surgical patients. The clinical definition of sepsis varied across studies but generally followed the ACCP/SCCM consensus conference definition’s clinical criteria closely [30].

**Table 1:** **Characteristics of studies included and summary of measures reported in validation studies** ^**a**^

**Authors, country, year [ref]**	**Sample population**	**Data years**	**Type of administrative database**	**Study size (n)**	**ICD version**	**Diagnostic coding field position**	**Reference/gold standard**	**Sn**	**Sp**	**PPV**	**NPV**
Cevasco *et al.*, USA, 2011 [19]	General surgical	2003 to 2007	Population-based, inpatient Veterans Affairs hospital	112	ICD-9-CM	Secondary	Medical chart review	–	–	53%	–
	General surgical	2005 to 2007	Population-based, inpatient community hospital	164	ICD-9-CM	Secondary	Medical chart review	–	–	41%	–
Gedeborg *et al.*, Sweden, 2007 [20]	ICU-specific	1994 to 1999	Population-based, inpatient	4,181	ICD-9^b^	Principal, secondary	ICU database	45.7%	97.5%	45.9%	97.5%
	ICU-specific	1994 to 1999	Population-based, inpatient	3,434	ICD-10^b^	Principal, secondary	ICU database	52.5%	92.6%	28.0%	97.3%
	ICU-specific and DI	1994 to 1999	Population-based, inpatient	4,181	ICD-9^b^	Principal, secondary	ICU database	17.2%	99.4%	56.1%	96.3%
	ICU-specific and DI	1994 to 1999	Population-based, inpatient	3,434	ICD-10^b^	Principal, secondary	ICU database	20.1%	98.4%	40.9%	95.7%
	ICU-specific	1994 to 1999	Population-based, inpatient	45	ICD-9^b^ ICD-10^b^	Principal, secondary	Sepsis clinical trial patients	42.2%	95.5%	7.4%	99.5%
	ICU-specific	1994 to 1999	Inpatient intensivist-coded ICU database	45	ICD-9^b^ ICD-10^b^	Principal, secondary	Sepsis clinical trial patients	51.5%	92.6%	5.6%	99.6%
	ICU-specific	1994 to 1999	Population-based, inpatient	4,181	ICD-9^c^	Principal, secondary	ICU database	43.0%	98.0%	49.7%	97.4%
	ICU-specific	1994 to 1999	Population-based, inpatient	3,434	ICD-10^c^	Principal, secondary	ICU database	43.0%	95.6%	–	–
	ICU-specific	1994 to 1999	Population-based, inpatient	4,181	ICD-9^b^	Principal	ICU database	31.7%	99.2%	63.4%	97.0%
	ICU-specific	1994 to 1999	Population-based, inpatient	3,434	ICD-10^b^	Principal	ICU database	21.8%	97.9%	36.4%	95.8%
	ICU-specific: CAS	1994 to 1999	Population-based, inpatient	4,181	ICD-9^b^	Principal	ICU database	51.1%	99.4%	66.7%	98.9%
	ICU-specific CAS	1994 to 1999	Population-based, inpatient	3,434	ICD-10^b^	Principal	ICU database	31.8%	99.0%	41.5%	98.3%
	ICU-specific CAP and DI	1994 to 1999	Population-based, inpatient	3,434	ICD-9^b^	Principal	ICU database	19.1%	99.8%	64.3%	98.2%
	ICU-specific CAS and DI	1994 to 1999	Population-based, inpatient	3,434	ICD-10^b^	Principal	ICU database	17.6%	99.4%	42.8%	97.9%
	ICU-specific CAS	1994 to 1999	Population-based, inpatient	3,434	ICD-9^c^	Principal	ICU database	47.9%	99.5%	70.3%	98.8%
	ICU-specific CAS	1994 to 1999	Population-based, inpatient	3,434	ICD-10^c^	Principal	ICU database	27.1%	99.0%	39.7%	98.2%
	ICU-specific CAS	1994 to 1999	Population-based, inpatient	45	ICD-9^c^ ICD-10^c^	Principal	Sepsis clinical trial patients	46.9%	97.4%	9.9%	99.7%
	ICU-specific CAS	1994 to 1999	Inpatient intensivist-coded ICU database	45	ICD-9^c^ ICD-10^c^	Principal	Sepsis clinical trial patients	31.2%	98.5%	10.9%	99.6%
Grijalva *et al.*, USA, 2008 [21]	Rheumatoid arthritis	1995 to 2004	Inpatient database	45	ICD-9-CM	Principal, secondary	Medical chart review	–	–	80%	–
Ibrahim *et al.*, Australia, 2012 [22]	General ICU	2000 to 2006	Inpatient database	1,645	ICD-10-AM	Principal	ICU database	44.1%	98.9%	88.2%	90.6%
	General ICU	2000 to 2006	Inpatient database	45	ICD-10-AM	Principal	ICU database	16.5%	99.8%	93.9%	86.8%
Iwashyna *et al.*, USA, 2014 [12]	General	2009 to 2010	Population-based, inpatient	111	ICD-9-CM Angus	All	Medical chart review	50.3%	96.3%	70.7%	91.5%
	General	2009 to 2010	Population-based, inpatient	111	ICD-9-CM Explicit	All	Medical chart review	9.3%	100%	100%	86.0%
	General	2009 to 2010	Population-based, inpatient	111	ICD-9-CM Martin	All	Medical chart review	16.8%	99.8%	97.6%	87.0%
Lawson *et al.*, USA, 2012 [23]	General surgical	2005 to 2008	Population-based claims data	13,410	ICD-9-CM	All	ACS-NSQIP inpatient surgical database	46.3%	94.0%	–	–
Madsen *et al.*, Denmark, 1998 [24]	General	1994	Population-based, inpatient	471	ICD-10, Danish version	Unknown	Bacteraemia database	5.9%	–	21.7%	–
Ollendorf *et al.*, USA, 2002 [25]	Severe sepsis clinical trial patients	No dates given	Population-based, inpatient claims	122	ICD-9-CM	All	Severe sepsis clinical trial patients	–	–	75.4%	–
Quan *et al.*, Canada, 2013 [26]	General surgical	2007 to 2008	Population-based, inpatient	117	ICD-10	Secondary	Medical chart review	–	–	9.8%	–
	General surgical	2007 to 2008	Population-based, inpatient	34	ICD-10	Secondary	Medical chart review	–	–	12.5%	–
Ramanathan *et al.*, USA, 2014 [27]	Surgical patients	2012 to 2013	Surgical inpatient	243	ICD-9-CM	All	Medical chart review	82.3%	78.3%	91.1%	62.1%
Schneeweiss *et al.*, USA, 2007 [28]	General	2001 to 2004	Population-based, inpatient	158	ICD-9-CM	Principal	Medical chart review	–	–	91%	–
Whittaker *et al.*, USA, 2013 [29]	ED admitted inpatients	2005 to 2009	Population-based, inpatient	1,735	ICD-9 (severe)	All	Medical chart review	20.5%	–	–	–
	ED admitted inpatients	2005 to 2009	Population-based, inpatient	1,735	ICD-9 (severe)	All	Medical chart review	47.2% (Angus)	–	–	–
	ED admitted inpatients	2005 to 2009	Population-based, inpatient	321	ICD-9 (shock)	All	Medical chart review	49.5%	–	–	–
	ED admitted inpatients	2005 to 2009	Population-based, inpatient	321	ICD-9 (shock)	All	Medical chart review	42.4%	–	–	–
	ED admitted inpatients	2005 to 2009	Population-based, inpatient	321	ICD-9 (shock)	All	Medical chart review	75.1% (Angus)	–	–	–

^a^CAS, Community-acquired sepsis (intensive care unit (ICU) admission within 48 hours); DI, Department of Infectious Disease patients; ICD, International Classification of Diseases; AM, Australian Modification; CM, Clinical Modification; ACS-NSQIP, American College of Surgeons National Surgical Quality Improvement Program; ED, Emergency Department; NPV, Negative predictive value; PPV, Positive predictive value; Sn, Sensitivity; Sp, Specificity. ^b^Sepsis wide criteria codes. ^c^Sepsis narrow criteria codes.

### Performance characteristics

Reference standard definitions included medical chart review, ICU registry database (both validated and not validated by ICU physicians), bacteraemia-specific registry database, surgical inpatient database and a cohort of patients who had been entered into severe sepsis clinical trials based on specified and defined inclusion criteria. A total of 38 ICD sepsis case definitions were tested with over 130 different ICD codes (see Table 2 for codes used in each study). The most commonly used codes were the ICD-9 codes 038.x (septicaemia, not otherwise specified (NOS)), 790.7 (bacteraemia, NOS) and 995.92 (severe sepsis) and the ICD-10 codes A40.x (streptococcal sepsis) and A41.x (other sepsis).

**Table 2:** **ICD version and ICD codes used in included studies** ^**a**^

**Author**	**ICD version**	**ICD codes used**
Cevasco *et al.*, USA, 2011 [19]	ICD-9-CM	0380, 0381, 03810, 03811, 03812, 03819, 0382, 0383, 78552, 78559, 9980, 99591, 99592, 03840, 03841, 03842, 03843, 03844, 03849, 0388, 0389
Gedeborg *et al.*, Sweden, 2007 [20]	ICD-9	*Sepsis, wide criteria*: 020–023, 027A, 032, 037, 040A, 041, 060, 061, 065, 071, 074C, 078G, 078H, 112X, 118, 590, 790H, 790 W
	ICD-10	*Sepsis, wide criteria*: A19–A36, A44.0, A49, A54.8, A69.2, A75–A79, B00.7, B00.9, B01.8, B01.9, B02.7–B02.9, B05.8, B05.9, B34.9, B38–B64, R50, T79.3, T81.3–T81.6, T83.6, T83.8, T84.5–T84.7, T85.7, T88.0, Y95
	ICD-9	*Sepsis, narrow criteria*: 036C–036E, 036X, 038, 084, 112 F, 117D, 286G, 999D
	ICD-10	*Sepsis, narrow criteria*: A02.1, A04.0–A04.3, A39–A41, A42.7, A48, A90–A99, B37.7, B38.7, B39.3, B40.7, B41.7, B42.7, B44.7, B45.7, B46.4, B95–B99, D65, T80.2
Grijalva *et al.*, USA, 2008 [21]	ICD-9-CM	003.1, 036.2, 785.52, 790.7, 038.x
Ibrahim *et al.*, Australia, 2012 [22]	ICD-10-AM	*Sepsis*: A40.0, A40.1, A40.2, A40.3, A40.8, A40.9, A41.0, A41.1, A41.2, A41.3, A41.4, A41.5, A41.52, A41.58, A41.8, A41.9
		*Cholecystitis*: K81.0, K83.0
		*Peritonitis*: K65.9
		*Pneumonia*: J13, J15.9, J18.0, J18.8, J18.9, J85.2
		*Perforation*: K22.3, K27.5, K63.1
Lawson *et al.*, USA, 2012 [23]	ICD-9-CM	038, 78552, 99591, 99592, 9980, 99859, 99931
Madsen *et al.*, USA, 1998 [24]	ICD-10, Danish version	A42.7, A41.3, A54.8, P36, P36.5, 36.4, P36.8, P36.2, P36.1, A02.1, A40.0, A40.2, A41.9, A40.8, O08.0, O85.9, A41.1, A41.2, A40.9, O75.3, A41.4, A41.5, P36.0, P36.3, P36.9, A41.0, A40.1, A40.3, A28.2, A41.8
Ollendorf *et al.*, USA, 2002 [25]	ICD-9-CM	038.3, 022.3, 790.7, 038.42, 038.49, 038.40, 038.41, 054.5, 036.2, 038.2, 038.43, 003.1, 038.8, 038.9, 020.2, 038.44, 038.1, 038.0
Schneeweiss *et al.*, USA, 2007 [28]	ICD-9-CM	*Bacteremia*: 038.-, 790.7
Quan *et al.*, Canada, 2013 [26]	ICD-10-CA	A40.0, A40.1, A40.2, A40.3, A40.8, A40.9, A41.0, A41.1, A41.2, A41.3, A41.4, A41.5, A41.8, A41.9, R57.8, T81.1
Iwashyna *et al.*, USA, 2014 [12]	ICD-9-CM	Angus positive:
		*Severe sepsis*: 995.92; *Septic shock*: 785.52;
		OR codes used to identify infection: 001, 002, 003, 004, 005, 008, 009, 010, 011, 012, 013, 014, 015, 016, 017, 018, 021, 022, 023, 024, 025, 026, 027, 030, 031, 032, 033, 034, 035, 036, 037, 038, 039, 040, 041, 090, 091, 092, 093, 094, 095, 096, 097, 098, 100, 101, 102, 103, 104, 110, 111, 112, 114, 115, 116, 117, 118, 320, 322, 324, 325, 420, 421, 451, 461, 462, 463, 464, 465, 481, 482, 485, 486, 491.21, 494, 510, 513, 540, 541, 542, 52.01, 562.03, 562.11, 562.13, 566, 567, 569.5, 569.83, 572.0, 572.1, 575.0, 590, 597, 599.0, 601, 614, 615, 616, 681, 682, 683, 686, 711.0, 730, 790.7, 996.6, 998.5, 999.3;
		AND acute organ dysfunction codes: 785.5, 458, 96.7, 343.3, 293, 348.1, 287.4, 287.5, 286.9, 286.6, 570, 573.4, 584
	ICD-9-CM	*Explicit code positive*: 995.92, 785.52
	ICD-9-CM	*Martin positive*: 038, 020.0, 112.5, 112.81; AND acute organ dysfunction codes: 785.5, 458, 96.7, 343.3, 293, 348.1, 287.4, 287.5, 286.9, 286.6, 570, 573.4, 584 OR 995.92 OR 785.52
Ramanathan *et al.*, USA, 2014 [27]	ICD-9-CM	995.91, 995.92, 785.52
Whittaker *et al.*, USA, 2013 [29]	ICD-9	995.92, 785.52, Angus coding method (see Iwashyna *et al*. [12])

^a^AM, Australian Modification; CA, Canadian edition; CM, Clinical Modification; ICD, International Classification of Diseases.

The validity of the ICD sepsis definitions varied greatly among studies. Seven of the twelve studies calculated Sn, and five studies calculated Sp. Sn ranged from 5.9% to 82.3% (median: 42.4%), and Sp ranged from 78.3% to 100% (median: 98.5%). The PPV was calculated in 10 of the 12 studies and ranged from 5.6% to 100% (median: 50%); NPV was provided in four studies and ranged from 62.1% to 99.7% (median: 97.4%) (Table 1).

One study [20] examined eighteen different case definitions using a ‘sepsis wide’ coded definition and a ‘sepsis narrow’ coded definition for both ICD-9 and ICD-10 codes. These coding algorithms were then compared. Among these case definitions, Sn varied from 17.2% to 52.5% (median: 37.0%) and Sp ranged from 92.6% to 99.8% (median: 98.5%) (Table 1).

After applying the standardized quality assessment checklist to each of the 12 included studies, the tallied scores ranged from 10 to 30, indicating variable quality among the studies (Table 3).

**Table 3:** **Quality assessment checklist of reporting criteria for validation studies of health administrative data** ^**a**^

	**Cevasco** ***et al.*** [19]	**Gedeborg** ***et al.*** [20]	**Grijalva** ***et al.*** [21]	**Ibrahim** ***et al.*** [22]	**Lawson** ***et al.*** [23]	**Madsen** ***et al.*** [24]	**Ollendorf** ***et al.*** [25]	**Schneeweiss** ***et al.*** [28]	**Quan** ***et al.*** [26]	**Iwashyna** ***et al.*** [12]	**Ramanathan** ***et al.*** [27]	**Whittaker** ***et al.*** [29]
1. Identify article as study of assessing diagnostic accuracy	1	1	1	1	1	1	1	1	1	1	1	1
2. Identify article as study of administrative data	1	1	1	1	1	1	1	1	1	1	1	1
3. State disease identification & validation as goals of study	1	1	1	1	1	1	1	1	1	1	1	1
*Methods: participants in validation cohort*												
4. Age	1	0	1	1	1	0	0	0	1	1	1	1
5. Disease	1	1	1	1	1	1	1	1	1	1	1	1
6. Severity	1	1	1	1	1	1	1	1	1	1	1	1
7. Location/jurisdiction	1	1	1	1	1	1	1	1	1	1	0	1
8. Describe recruitment procedure of validation cohort	1	0	1	1	1	1	0	1	1	1	1	1
9. Inclusion criteria	1	0	1	1	1	1	0	1	1	1	1	1
10. Exclusion criteria	1	1	1	1	1	1	0	1	1	1	0	1
11. Describe patient sampling (random, consecutive, all, etc.)	1	1	1	1	1	1	0	1	1	1	1	1
12. Describe data collection	1	1	1	1	1	1	0	1	1	1	1	1
13. Who identified patients and did selection adhere to patient recruitment criteria	1	0	1	1	1	1	0	1	1	1	1	1
14. Who collected data	1	0	1	1	1	1	0	1	1	1	1	1
15. *A priori* data collection form	1	0	1	1	1	1	1	1	1	1	0	1
16. Disease classification	1	1	1	1	1	1	1	1	1	1	1	1
17. Split sample (that is, revalidation using a separate cohort)	0	0	0	0	0	U	0	0	0	0	0	0
*Test methods*												
18. Describe number, training and expertise of persons reading reference standard	1	1	1	1	1	0	0	1	1	1	1	1
19. If more than one person reading reference standard, quote measure of consistency (for example, κ)	1	0	1	N/A	N/A	0	0	N/A	0	0	0	0
20. Blinding of interpreters of reference standard to results of classification by administrative data (for example, chart abstractor blinded to how that chart was coded)	U	1	1	U	0	0	0	0	1	1	0	1
*Statistical methods*												
21. Describe methods of calculating diagnostic accuracy	1	1	1	1	1	1	0	1	1	1	0	1
*Results: participants:*												
22. Report when study done, start/end dates of enrolment	1	1	1	1	1	1	0	1	1	1	1	1
23. Describe number of people who satisfied inclusion/exclusion criteria	1	1	1	1	1	1	1	1	1	1	1	1
24. Study flow diagram	0	0	0	1	0	0	0	0	1	0	0	0
*Test results:*												
25. Report distribution of disease severity	1	1	1	1	0	1	0	0	1	1	1	1
26. Report cross-tabulation of index tests by results of reference standard	1	1	1	1	0	1	0	0	1	0	0	0
27. Report at least four estimates of diagnostic accuracy	0	1	0	1	1	0	0	0	0	1	1	0
*Diagnostic accuracy measures reported*												
28. Sensitivity	0	1	0	1	1	1	0	0	0	1	1	1
29. Specificity	0	1	0	1	1	0	0	0	0	1	1	0
30. PPV	1	0	1	1	1	1	1	1	1	1	1	0
31. NPV	0	0	0	1	1	0	0	0	0	1	1	0
32. Likelihood ratios	0	1	0	0	0	0	0	0	0	0	0	0
33. κ	0	0	0	0	0	0	0	0	0	0	0	0
34. Area under the ROC curve/C-statistic	0	0	0	0	0	0	0	0	0	0	0	0
35. Accuracy/agreement	0	0	0	0	0	0	0	0	0	0	0	0
36. Other (specify)	0	0	0	0	0	0	0	0	0	0	0	0
37. Report accuracy for subgroups (for example, age, geography)	0	1	0	0	0	0	0	1	1	0	0	1
38. If PPV/NPV reported, does the ratio of cases/controls of validation cohort approximate prevalence of condition in the population?	1	1	N/A	N/A	1	N/A	N/A	N/A	N/A	0	0	N/A
39. Report 95% CI for each diagnostic measure	1	1	1	1	1	1	0	0	1	1	1	1
*Discussion*												
40. Discuss the applicability of the validation findings	1	1	1	1	1	1	0	1	1	1	1	1
Total score	27	25	27	30	28	24	10	22	28	29	24	26

^a^CI, Confidence interval; N/A, Not applicable; NPV, Negative predictive value; PPV, Positive predictive value; ROC, Receiver operating characteristic. Yes = 1; No = 0; U = Unsure. Adapted from Benchimol *et al*. [17].

## Discussion

In this review, we identified and summarized the published literature evaluating and validating ICD-9 and ICD-10 codes used to identify sepsis in administrative databases. We identified 12 studies that met all eligibility criteria for this systematic review and found large variations in terms of the scope of ICD codes used and the estimates of validity among studies. All studies validated inpatient data, and the majority of the studies showed that ICD codes defining a diagnosis of sepsis in administrative data are highly specific but lack Sn. In 10 of the 12 studies, Sn was low (<53%), even in cases of altering study characteristics [20]. A reasonable conclusion is that sepsis is largely undercoded in administrative data using ICD-9 or ICD-10 coded case definitions, regardless of study characteristics. However, the high Sp and NPV do mean that few false-positives would be present in such a dataset.

The heterogeneity seen among the studies in coding accuracy, especially with respect to Sn and PPV, may be due to multiple factors, including the number of codes used, the version of ICD used, the sample population, the reference standard comparison used and the type of administrative data. For instance Gedeborg *et al.* [20] applied the same ICD-9 and ICD-10 coding algorithms to different patient populations, including ICU patients with community-acquired sepsis and infectious disease department patients, and tested these against two different reference standard definitions (sepsis clinical trial patients and patients from an ICU-specific coded database). They showed the data accuracy to have large variations that were dependent on the patient population being studied and reference standard used. Not surprisingly, limiting the sample population to one in which an infectious disease service was consulted during the patient stay actually decreased the Sn by 28.5% while only increasing the Sp by 1.9%. It has also been reported that severe sepsis is poorly documented outside the ICU, although in one study sepsis was commonly found on non-ICU medical wards [31], suggesting that the accuracy of diagnostic codes may be substantially impacted, depending on the population selected or the criteria used to define the population.

Validity is also dependent on diagnostic coding field location (primary or secondary or all). Cevasco *et al*. [19] examined a population-based inpatient database but restricted the sepsis diagnostic code to a secondary coding field position in two separate populations, resulting in lower PPV values (43% for Veterans Affairs patients and 51% for community hospital patients). Grijalva *et al*. [21] restricted the population to a highly specific patient sample (rheumatoid arthritis patients) and examined only five ICD-9-CM codes; however, they allowed the coding field position to be either primary or secondary, which resulted in a PPV of 80%. Gedeborg *et al*. [20] performed multiple comparisons using primary or both primary and secondary code field positions. They reported consistently high Sn estimates when both the primary and secondary coding field positions were included. The primary coding field is normally designated for the condition that contributed the most to a patient’s length of stay or was the main reason for admission (depending on country). Thus, sicker patients presenting with severe sepsis or septic shock are more likely to be captured using the primary diagnosis alone. A further limitation of severity level coding is reflected in the organ dysfunction codes used to identify severe sepsis, as these diagnostic codes would most likely be recorded in the secondary code field positions. In none of the studies were any particular organ dysfunction codes validated or the coding field positions examined.

The variation in diagnosing sepsis alone translates to variable recording of the diagnosis in the medical record. O’Malley *et al*. [32] described the patient trajectory from admission to discharge and the process of recording the admitting diagnosis to the assignment of an ICD code post-discharge. A suggested error when a physician records a diagnosis in the medical record is based on the variance across terms and language used to describe the disease and/or reporting of an infection without concomitant reporting of systemic inflammation or associated organ dysfunction. Peoze *et al*. [33] examined how a physician’s awareness and attitude towards the diagnosis of sepsis impacted the recording of sepsis. They reported that 46% of the time in the case of sepsis, the cause of death was incorrectly recorded as due to another disease. Assunção *et al*. [34] found that sepsis was most frequently misdiagnosed, up to 66.5% of the time, as infection without clinical and laboratory signs of inflammatory response. Therefore, low case capture of sepsis may also be due to the capacity of practicing physicians to recognize and report clinical cases of systemic inflammatory response syndrome, sepsis, severe sepsis and septic shock in the medical record. No study examined the expertise of the coders or the impact of physician documentation on the selected codes.

The results of this systematic review should raise a question about whether reliable research on sepsis can be performed using administrative data. On the basis of the findings of our review, hospital discharge abstract data alone are an insufficient source for researchers to examine sepsis incidence accurately or for surveillance. However, administrative data and ICD coding algorithms could still be used to examine risk factors for the development of sepsis or outcomes. In these studies, a high Sp with a reduced Sn may suffice to minimize the number of false-positive cases, with a caveat being a limitation that these studies may include a subset of more easily defined and/or recognized cases or a more severe form of sepsis.

The complexity which makes up the clinical entity of sepsis has led to a significant effort over the past 20-plus years to standardize clinical and laboratory diagnostic criteria and definitions [10,30,35,36]. Although designed primarily for clinical use, these definitions have led to practical applications for other research, including health care quality and utilization improvement initiatives and surveillance. Particularly for surveillance, one of the purposes is to monitor disease prevalence over a span of years and forecast future trends. Thus, the trend is related to the stability of the data validity in the observation period, regardless of the level of validity. That said, administrative data are still an invaluable resource to monitor sepsis, although it does not capture the same amount of clinical detail that an Electronic Medical Record (EMR) does. Other advantages, such as wide geographical coverage, a population-based capture of nearly every contact with the health care system and the overall cost-effectiveness [6], make administrative data a lucrative source of health information. Administrative data cannot replicate the complex myriad of the clinical criteria comprising sepsis; therefore, translating this clinical definition into coded data and evaluating the validity of the coding of sepsis in administrative data are crucial.

Although a desired definition with Sn and Sp of 100% would be ideal, modifying and optimizing the data definition to capture sepsis as accurately as possible, with Sn falling above 75%, similar to that of other hospital-acquired infections internationally [37] and for non-communicable diseases such as hypertension [38] and diabetes [39], should be the ultimate goal. Improving the quality of administrative health data and increasing the case capture and validity of sepsis could be accomplished through a number of simple strategies, such as (1) improved physician documentation, including documenting sepsis in the front pages of the chart to get the attention of coders; (2) having a specialized coding procedure for ICU patients, perhaps including specific training of health care coders to improve familiarity with the case mix of patients and conditions that are more prevalent in the ICU to increase Sn and case capture; and (3) for those countries in which a limited number of diagnostic coding fields exist, there should be at least eight coding fields for diagnosis to capture conditions such as sepsis [40]. These strategies can be used in combination with data linkage to other data sources such as laboratory, pharmacy or microbiology data and the EMRs, and with clinical factors such as heart rate, respiratory rate, body temperature, white blood cell count and markers of organ dysfunction, to try to incorporate the key characteristics of sepsis defined and listed in the ACCP/SCCM definitions [30]. Both improving the definition of sepsis and making it comparable across national and international jurisdictions is of the utmost importance to continue improving the understanding of how quality of sepsis care is impacting the incidence and outcomes of the disease.

There are limitations to this systematic review. The search strategy was limited to only studies published in English, and a grey literature search was not conducted. The target of the study was ICD codes used for sepsis specifically. Because sepsis itself is difficult to diagnose and has a range of clinical presentations, there is a possibility that validation studies examining only these other conditions and not sepsis specifically may have been missed. Publication bias in validation studies may also be a concern, as authors may report only better-performing case definitions and may not publish less well-performing case definitions with very low diagnostic accuracy. However, our systematic review included studies with very low values for case definitions, and therefore there is little concern that publication bias has occurred.

## Conclusions

Validated case definitions for sepsis have been reported with varying degrees of accuracy in studies using administrative data. Sepsis remains one of the top causes of death, specifically in the ICU, and as more researchers are utilizing administrative data to study sepsis outcomes and health services associated with care, an accurate ICD coded case definition is needed. Future studies are warranted to optimize the ascertainment of sepsis in administrative data, whether by testing new enhanced definitions, by optimizing physician documentation and/or by considering data linkage..

## Key messages

- Sepsis is undercoded in administrative data using ICD-9- and ICD-10-based case definitions.
- There is high heterogeneity across studies for coding sepsis in administrative data, which is dependent on the ICD codes used, the population studied, the criteria used to define sepsis and the diagnostic coding position, to name a few.
- To improve the capture of true sepsis cases in administrative data, strategies should be considered that include data linkage, improving physician documentation, implementing specialized coding procedures for ICU patients and the use of at least eight coding fields for diagnosis to capture complex conditions such as sepsis.

## Additional files

Additional file 1: Table S1. Search strategy terms used in Ovid, MEDLINE and Embase databases. Additional file 2: Table S2. List of excluded articles and reason for exclusion.

## Abbreviations

ACCP: American College of Chest Physicians
ACS: American College of Surgeons
CAS: Community-acquired sepsis
DI: Department of Infectious Disease
ED: Emergency department
EMR: Electronic Medical Record
ICD: International Classification of Diseases
ICD-9: International Classification of Diseases, Ninth Revision
ICD-10: International Classification of Diseases, Tenth Revision
ICD-10-AM: International Classification of Diseases, Tenth Revision, Australian Modification
ICD-10-CA: International Classification of Diseases, Tenth Revision, Canadian edition
ICD-9-CM: International Classification of Diseases, Ninth Revision, Clinical Modification
ICU: Intensive care unit
MeSH: Medical Subject Headings
NPV: Negative predictive value
NSQIP: National Surgical Quality Improvement Program
PPV: Positive predictive value
SCCM: Society of Critical Care Medicine
Sn: Sensitivity
Sp: Specificity
