# Supplementary material for: Validity of administrative data in recording sepsis: a systematic review
Source: Crit Care. 2015 Apr 6;19(1):139. doi: 10.1186/s13054-015-0847-3 (PMC4403835; doi:10.1186/s13054-015-0847-3)
Supplement: Additional file 1: Table S1. — Search strategy terms used in Ovid, MEDLINE and Embase databases. [file 13054_2015_847_MOESM1_ESM.docx]

| **Category** | **Key Words** |
| --- | --- |
| Sepsis | exp Sepsis/ OR Sepsis.mp OR Septicemia.mp OR exp Shock, Septic/ OR shock, septic.mp OR Septic shock.mp OR blood poisoning*.mp OR bloodstream infection.mp OR exp Bacteremia/ OR Bacteremia.mp OR exp Fungemia/ OR Fungemia.mp OR exp Parasitemia/ OR Parasitemia.mp OR exp Viremia/ OR Viremia.mp |
| AND | |
| Coding | exp Health Services Research/ OR health services research.mp OR administrative data.mp OR exp Hospital Records/ OR hospital records.mp OR exp Medical Records/ OR medical record*.mp OR health information.mp OR surveillance.mp OR physician claims.mp OR claims.mp OR hospital discharge.mp OR coding.mp OR codes.mp OR exp "International Classification of Diseases"/ OR international classification of disease.mp OR ICD.mp OR ICD9.mp OR ICD-9.mp OR ICD-9-CM.mp OR ICD10.mp OR ICD-10.mp OR ICD-10-CM.mp OR ICD-10-CA.mp |
| AND | |
| Validation | (validity OR validation OR case definition OR algorithm OR agreement OR accuracy OR sensitivity OR specificity OR positive predictive value OR negative predictive value).mp |
