# Supplementary material for: Validity of administrative data in recording sepsis: a systematic review
Source: Crit Care. 2015 Apr 6;19(1):139. doi: 10.1186/s13054-015-0847-3 (PMC4403835; doi:10.1186/s13054-015-0847-3)
Supplement: Additional file 2: Table S2. — List of excluded articles and reason for exclusion. [file 13054_2015_847_MOESM2_ESM.docx]

| **No.** | **Author** | **Year** | **Reason for Exclusion** |
| --- | --- | --- | --- |
|  | Abrusci T | 2011 | Does not validate sepsis ICD codes |
|  | Ahishakiye D | 2009 | No ICD code validation |
|  | Aitken LM | 2011 | No ICD code validation |
|  | Al-Hadeedi S | 1991 | No ICD code validation, published before 1992 |
|  | Al-Juaid A | 2012 | No ICD code validation |
|  | Alavi A | 2011 | No ICD code validation |
|  | Anaya DA | 2003 | No ICD code validation |
|  | Angyo IA | 2001 | No ICD code validation |
|  | Arnold FW | 2003 | No ICD code validation |
|  | Aube H | 1992 | No ICD code validation |
|  | Bacli C | 2003 | No ICD code validation |
|  | Bahrami S | 2010 | No ICD code validation |
|  | Balaban U | 2012 | No ICD code validation |
|  | Banatvala N | 1997 | Does not validate sepsis ICD codes |
|  | Bang AT | 2005 | No ICD code validation, only a diagnostic system |
|  | Barber C | 2013 | Not original research-review |
|  | Bavdekar SB | 2005 | No ICD code validation |
|  | Beekmann SE | 2005 | No ICD code validation |
|  | Begier EM | 2005 | No ICD code validation, Does not validate sepsis ICD codes |
|  | Bell LM | 1992 | No ICD code validation |
|  | Bellini C | 2007 | No ICD code validation |
|  | Bennett NJ | 2007 | No ICD code validation- only MRSA infection |
|  | Bhatt SP | 2007 | Not original research |
|  | Bhattarai J | 2011 | No ICD code validation |
|  | Bouam S | 2003 | No ICD code validation |
|  | Bouletreau A | 1999 | No ICD code validation |
|  | Bouza E | 2011 | No ICD code validation |
|  | Brown C | 2009 | No ICD code validation |
|  | Brown SM | 2009 | No ICD code validation |
|  | Buchanan-Chell M | 1992 | No ICD code validation |
|  | Capp R | 2011 | No ICD code validation |
|  | Carnahan RM | 2012 | Not original research |
|  | Cevasco M | 2011 | Does not validate sepsis ICD codes – CVC-related BSIs |
|  | Chiu D | 2011 | No reference standard comparison and no administrative data |
|  | Cho YK | 2011 | No ICD code validation |
|  | Choe Y | 2011 | No reference standard comparison |
|  | Chowdhury HR | 2010 | No administrative data used |
|  | Cremer OL | 2014 | Not original research-editorial |
|  | Damjanovic V | 1995 | Not original research |
|  | Davis BH | 2005 | No ICD code validation |
|  | De Prost N | 2010 | No ICD code validation |
|  | De Wals P | 1984 | Published before 1992, used ICD codes, version unknown |
|  | Elramah M | 2012 | No reference standard comparison |
|  | Emori TG | 1998 | No ICD code validation, Does not validate sepsis ICD codes |
|  | Esper AM | 2006 | Does not validate sepsis codes |
|  | Ferreira J | 2014 | No ICD code validation |
|  | Fischer JE | 2005 | Not original research, No ICD code validation |
|  | Fontela PS | 2013 | Does not validate sepsis ICD codes |
|  | Fry DE | 2007 | No reference standard comparison |
|  | Gerald J | 2011 | Abstract - used ICD-9 as reference standard |
|  | Golden WE | 1995 | No ICD codes validation |
|  | Graham PL | 2004 | No ICD code validation |
|  | Guasticchi G | 2009 | No ICD code validation |
|  | Guevara RE | 1999 | Does not validate sepsis ICD codes -pneumococcal pneumonia |
|  | Hayward J | 1986 | Published before 1992 |
|  | Horng S | 2012 | Abstract available with little information |
|  | Hsu LY | 2008 | Published before 1992 |
|  | Jaimes F | 2003 | No ICD code validation, Does not validate sepsis ICD codes – only SIRS |
|  | Juskewitch JE | 2012 | No ICD code validation, No administrative data |
|  | Lagu T | 2011 | No ICD code validation -Looking at sepsis severity not ability of codes to capture sepsis |
|  | Lagu T | 2012 | No ICD code validation |
|  | Leal J | 2008 | Not original research |
|  | Lesher L | 2009 | Does not validate sepsis ICD codes- only TSS definition |
|  | Leth RA | 2006 | No ICD code validation |
|  | Linde-Zwirble WT | 2011 | No reference standard comparison |
|  | Liu FX | 2010 | No measures of accuracy for ICD codes |
|  | McIntosh EDG | 2003 | No measures of accuracy for ICD codes |
|  | Misset B | 2009 | No reference standard comparison |
|  | Modi N | 2013 | No ICD code validation |
|  | Moehring RW | 2009 | Does not validate sepsis ICD codes, looking at HAIs |
|  | Moore LJ | 2013 | No ICD codes |
|  | Patrick SW | 2010 | Does not validate sepsis ICD codes-Only CLABSI |
|  | Ramanathan R | 2013 | Does not validate sepsis ICD codes -Abstract |
|  | Romano PS | 2003 | Does not validate sepsis ICD codes |
|  | Ruhnke GW | 2009 | No ICD code validation |
|  | Stevenson KB | 2008 | Does not validate sepsis codes - looking at HAP, HAI |
|  | Tabak YP | 2007 | No ICD code validation |
|  | Thompson DS | 2003 | No administrative data, tool evaluation |
|  | Verelst S | 2010 | No ICD code validation |
|  | Wallgren U | 2011 | ICD codes used as reference standard, No validation |
|  | Watson RS | 2012 | Not original research |
|  | Weiss SL | 2012 | No reference standard comparison |
|  | Yokoe DS | 1998 | No ICD code validation |
